# Evaluating the hidden potential of deashed biochar in mitigating salinity stress for cultivation of fenugreek

**DOI:** 10.1038/s41598-023-49063-8

**Published:** 2024-01-02

**Authors:** Shoucheng Huang, Ping Huang, Misbah Hareem, Muhammad Tahzeeb-ul-Hassan, Uzma Younis, Khadim Dawar, Shah Fahad, Saleh H. Salmen, Mohammad Javed Ansari, Subhan Danish

**Affiliations:** 1 College of Life and Health Science, Anhui Science and Technology University, Fengyang, 233100 China; 2 College of Chemistry and Materials Engineering, Anhui Science and Technology University, Bengbu, 233000 China; 3 Department of Environmental Sciences, The Woman University Multan, Multan, Punjab Pakistan; 4 Charles Darwin University, Casurina Campus, Darwin, NT 0812 Australia; 5 Botany Department, The Islamia University of Bahawalpur, Rahim Yar Khan, Punjab Pakistan; 6 Department of Soil and Environmental Science, The University of Agriculture Peshawar, Peshawar, Pakistan; 7 Department of Agronomy, Abdul Wali Khan University Mardan, Mardan, 23200 Khyber Pakhtunkhwa Pakistan; 8 Department of Botany and Microbiology, College of Science, King Saud University, PO Box 2455, 11451 Riyadh, Saudi Arabia; 9 Department of Botany, Hindu College Moradabad (Mahatma Jyotiba Phule Rohilkhand University Bareilly), Moradabad, 244001 India; 10 Department of Soil Science, Faculty of Agricultural Sciences and Technology, Bahauddin Zakariya University, Multan, Punjab Pakistan

**Keywords:** Plant sciences, Plant stress responses, Salt

## Abstract

Soil salinity, the second most prominent cause of land degradation after soil erosion, has posed a persistent challenge to agriculture. Currently, approximately 1 billion hectares of Earth's land surface, equivalent to 7%, are affected by salinity. While biochar has proven effective in mitigating salinity stress, the specific role of deashed biochar in salinity mitigation has not been thoroughly explored. Therefore, this study was conducted to investigate the impact of four levels of deashed biochar (0%, 0.4%, 0.8%, and 1.2%) on the growth and physiological attributes of Fenugreek under both non-saline conditions (2.54 dS/m EC) and salinity stress conditions (5.46 dS/m EC). The results revealed a notable enhancement in various parameters under salinity stress. Compared to the control, the application of 1.20% deashed biochar led to a significant increase in shoot fresh weight (30.82%), root fresh weight (13.06%), shoot dry weight (17.43%), root dry weight (33.44%), shoot length (23.09%), and root length (52.39%) under salinity stress. Furthermore, improvements in internal CO2 concentration (9.91%), stomatal conductance (15.49%), photosynthetic rate (25.50%), and transpiration rate (10.46%) were observed, validating the efficacy of 1.20% deashed biochar in alleviating salinity stress. The study also demonstrated a significant decrease in the activities of oxidative stress markers such as peroxidase (POD), superoxide dismutase (SOD), catalase (CAT), ascorbate peroxidase (APX), electrolyte leakage, and malondialdehyde (MDA). Simultaneously, there was an increase in the concentrations of essential nutrients, namely nitrogen (N), phosphorus (P), and potassium (K), in both shoot and root tissues. These findings collectively suggest that deashed biochar, particularly at a concentration of 1.20%, is recommended for achieving enhanced crop production under conditions of salinity stress.

## Introduction

Soil salinity, the second most significant cause of land degradation following soil erosion, has long posed a formidable challenge to agriculture^1^. Presently, approximately 1 billion hectares of the Earth's land surface—equivalent to 7%—are affected by salinity^2^. Globally, an alarming rate of 2000 hectares of arable land is lost each day due to salinity^3^. This phenomenon results in a substantial 10–25% reduction in crop yields, and under severe conditions, it leads to desertification^3^. Consequently, adopting imperative measures to mitigate the impact of salinity is crucial, not only to preserve arable land but also to sustainably enhance crop production^4^. This undertaking is vital to ensuring food security for the ever-growing global population.

To mitigate the impact of soil salinity on crops, various measures are implemented, such as enhanced irrigation^2^, efficient water management^5^, cultivation of salt-tolerant crops^6^, crop rotation^7^, and soil amendments^8^. These strategies aim to minimize salinity effects and preserve crop yields. However, despite their potential benefits, addressing soil salinity presents challenges. High costs, technical intricacies, variable effectiveness of salt-tolerant crops, and energy-intensive desalination for irrigation^6,9^.

Biochar, produced through the pyrolysis of organic materials, offers a promising solution for mitigating soil salinity in agriculture^1,10,11^. Its high cation exchange capacity effectively immobilizes salt ions, reducing overall soil salinity^12^. Additionally, biochar's porous structure improves water retention, preventing rapid evaporation in saline soils. It fosters microbial activity, contributing to nutrient cycling and enhanced soil fertility^13^. Kanwal et al.^14^found improvements in wheat seed germination and growth with 1% and 2% biochar applications under four concentrations of NaCl solution (0 mM, 50 mM, 100 mM, and 150 mM). Kul et al.^13^ found an increase of 113.7%, 74.8%, 24.9%, 115.5%, and 62.5% in tomato shoot fresh weight, shoot dry weight, number of leaves, root fresh weight, and root dry weight with 10% biochar application in saline soil (10 mM).

Biochar that has undergone deashing refers to biochar from which ash content has been removed or significantly reduced. This process involves eliminating or reducing the inorganic mineral components found in the original biochar, such as salts and other ash-forming materials. Deashing biochar, by reducing or removing inorganic minerals, enhances purity and tailors the material for specific applications like agriculture and environmental remediation. Sun et al.^15^ conducted a study examining the sorption characteristics of high-mineral biochar in contrast to low-mineral biochar. Their findings revealed that soils enriched with deashed biochar exhibited a sorption affinity that did not align with the anticipated level based on the sorption properties observed in fresh biochar. Zhong et al.^16^ found that treating biochar with acid increased its organic carbon, oxygen, aromaticity, and oxygen-containing functional groups. Additionally, it enhanced the surface area and pore volume of the biochar. In another study, Chen, and team^17^ observed that biochar subjected to deashing exhibited the highest level of graphitization, making it resistant to environmental degradation and beneficial for carbon sequestration. This deashed biochar also possessed the highest concentration of oxygen-containing functional groups and aliphaticity, which are easily degradable in the environment, making it a potential carbon source for soil organisms' growth. Ibrahim et al.^18^found that removing ash from biochar heightened the strength of its functional groups and changed its surface structure. When de-ashed bamboo biochar and de-ashed cow manure biochar were used in soils with manure, they increased NH_4_^+^-N retention by 34.5% and 6.8%, respectively, compared to using raw biochar.

Fenugreek (Trigonella foenum-graecum) is an important medicinal and culinary herb known for its aromatic seeds and leaves. When exposed to high salt levels in the soil or irrigation water, fenugreek plants can experience detrimental effects on their physiology and productivity^21–24^

The functioning of deashed biochar in saline soil has not been thoroughly investigated. Consequently, our hypothesis posits that deashed biochar can alleviate the detrimental impact of salinity on fenugreek productivity. Our primary objectives were to: (i) assess the impact of deashed biochar on crop performance both in the absence and presence of salinity stress, (ii) evaluate nutrient levels in the roots and shoots of plants under non-saline and saline conditions, and (iii) examine the activity of antioxidants specifically under salinity stress.

## Material and methods

### Soil sampling and characteristics

Soil samples were collected from the vicinity of the Chenab River, specifically located in Multan, Punjab, Pakistan, with geographical coordinates of approximately 30°19′20.8′′ N 71°24′50.4′′ E. The soil found at the experimental site displayed a dark yellowish-brown coloration, contained a moderate amount of calcium carbonate, exhibited a somewhat limited structural organization, showed the presence of a Cambic subsurface horizon, and was identified by the presence of an Ochric epipedon^19^.

### Experimental design

The deashed biochar levels (0, 0.4, 0.8, 1.2%) and salinity stress levels (no salinity stress and salinity stress) treatment was applied according to a Complete Randomized Design (CRD). The pots were divided into eight treatment groups, with four replicates per treatment. The metrological data of the experiment duration is provided in Table 1.

**Table 1:** Climatic data of the experimental site.

Month	Maximum temperature (°C)	Minimum temperature (°C)	Relative humidity (%)	Rainfall (mm)	Sunshine (h)
October-2021	33.80	20.40	57.70	0.00	7.50
November-2021	28.10	13.20	48.10	0.00	6.30
December 2021	21.70	7.10	48.60	0.00	4.90
January 2022	19.60	7.20	48.90	56.60	5.90
February 2022	23.90	10.70	48.00	0.00	8.00
March 2022	33.60	17.50	49.10	0.50	8.30

### Soil characterization

Before beginning the experiment, soil samples were procured randomly from the specified collection site. A total of eight samples were collected and then combined to form a composite sample, which was used for the initial soil characterization. The soil texture analysis was conducted using a hydrometer in accordance with the USDA textural triangle method^20^. To determine soil pH and electrical conductivity (EC), pre-calibrated pH and EC meters were utilized. Soil and deionized water mixtures were prepared in the ratios of 1:1 and 1:10, respectively, to facilitate the measurement of pH and EC^21,22^. The assessment of soil organic matter content was conducted using the potassium dichromate methodology. The final values were determined through a titration process involving ferrous ammonium sulfate^23^. Total soil nitrogen was quantified utilizing Kjeldhal's distillation method^24^. For the analysis of available phosphorus and potassium, Olsen and ammonium acetate extracting reagents were used^25,26^. The final values for phosphorus were determined by measuring the samples with a spectrophotometer at a wavelength of 880 nm, and the potassium content was assessed using a flame photometer. Pre-experimental data regarding soil characteristics is presented in Table 2.

**Table 2:** Pre-experimental soil, biochar, and irrigation characteristics.

Soil	Values	Biochar	Values	Irrigation	Values
pH	8.24	pH	7.05	pH	6.35
SOC (%)	0.40	EC*e* (dS/m)	1.45	EC (µS/cm)	115
TN (%)	0.020	Volatile matter (%)	40	Carbonates (meq/L)	0.00
EP (mg/kg)	6.12	Fixed carbon (%)	60	Bicarbonates (meq/L)	4.12
AK (mg/kg)	147	TN (%)	0.15	Chloride (meq/L)	0.10
Sand (%)	25	TP (%)	0.05	Ca + Mg (meq/L)	3.20
Silt (%)	40	TK (%)	0.31	Sodium (mg/L)	93
Clay (%)	35	Surface area (m^2^/g)	450		
Texture	Clay loam	CEC (meq/100 g)	500		

*TN* total nitrogen, *EP* extractable phosphorus, *AK* available potassium, *CEC* cation exchange capacity.

### Deashed biochar preparation

In the preparation of the deashed biochar used in this study, cotton sticks were obtained as raw feedstock. The biomass material was pyrolyzed at 430 °C under anaerobic conditions to produce biochar. Subsequently, the raw biochar was washed with deionized water to remove water-soluble ash components. The washing process included soaking the biochar in water and repeated filtration (6 times) to separate the biochar from the liquid. After washing, the biochar was dried to remove excess moisture. The dried biochar was then sieved (< 2 mm sieve) to obtain a uniform particle size distribution. The characteristics of biochar are provided in Table 1.

### Treatment plan

Deashed biochar was applied at four different levels: 0%, 0.3%, 0.6%, and 0.9% (by weight). The biochar was added to the soil in each pot according to the specific treatment requirements. Two salinity levels were considered: 2.54 dS/m and 5.46 dS/m. These salinity levels were achieved by adding the appropriate amount of salt to the irrigation water for each treatment group. The eight treatment groups were labeled as follows: Control (0%DB) + 2.54 dS/m, 0.3% deashed biochar + 2.54 dS/m, 0.6% % deashed biochar + 2.54 dS/m, 0.9% deashed biochar + 2.54 dS/m, SS (0% deashed biochar) + 5.46 dS/m, 0.3% deashed biochar + 5.46 dS/m, 0.6% % deashed biochar + 5.46 dS/m and 0.9% % deashed biochar + 5.46 dS/m. Biochar was applied in soil on a w/w basis.

### Fertilizers

During the sowing process, 0.03 g of nitrogen (equivalent to 0.074 g of urea per 5 kg of soil) and 0.050 g of P_2_O_5_ (equivalent to 0.31 kg of superphosphate per 5 kg of soil) were added to each pot. Furthermore, a single spray of NPK (20:20:20) fertilizer at a rate of 5 g/L of water was applied approximately 20 days after sowing.

### Irrigation

We maintained 65% field capacity (FC) in the pots by irrigating them, using a moisture meter (YIERYI 4 in 1; Shenzhen, Guangdong Province, China) for precise control^27^.

### Harvesting

After 45 days of sowing, plants were harvested for data collection. Shoot and root fresh weights were determined soon after harvesting on analytical grade balance. For dry weight, samples were heated at 70 °C for 48 h in the oven. Finally, dry weight was recorded by using analytical grade balance.

### Chlorophyll contents and carotenoids

The extraction process utilized a solution consisting of 80% acetone. To determine the concentrations of chlorophyll a and b, the absorbance was measured at 663 nm and 645 nm, respectively^28^. Carotenoids were assessed by measuring the absorbance at 470 nm. Similarly, anthocyanin measurements were taken at a wavelength of 530 nm.

$${\mathrm{Chlorophyll}} \, a \,\left(\frac{{\text{mg}}}{{\text{g}}}\right) = \frac{\left(12.7 \times {\mathrm{A}}663\right)- \left(2.69 \times {\mathrm{A}}645\right)\times {\text{V}}}{1000 \times {\text{W}}}$$

$$\mathrm{Chlorophyll } \,b \, \left(\frac{{\text{mg}}}{{\text{g}}}\right)=\frac{\left(22.9 \times \mathrm{ A}645\right)- \left(4.68 \times \mathrm{ A}645\right)\times {\text{V}}}{1000 \times {\text{W}}}$$

$$\mathrm{Total \, Chlorophyll }\left(\frac{{\text{mg}}}{{\text{g}}}\right)= 20.2\left(\mathrm{OD \,}645\right)+8.02\left(\mathrm{OD }663\right)\times {\text{V}}/1000 \,({\text{W}})$$

$$\mathrm{Carotenoids }\left(\frac{{\text{mg}}}{{\text{g}}}\right)={\text{OD}}480+0.114 \,\left({\text{OD}}663\right)-0.638 \,({\text{OD}}645)$$

$$\mathrm{Anthocyanin }\left(\frac{{\upmu {\rm mol}}}{{\text{ml}}}\right)=\left(0.08173 \times \mathrm{OD \,}537\right)-\left(0.00697 \times 0{\text{D}}645\right)-(0.002228\times {\text{OD}}663)$$

### Gas exchange attributes

The CI-340 Photosynthesis system, manufactured by CID, Inc. USA, was employed as an infrared gas analyzer to measure the stomatal conductance, net photosynthetic rate, and net transpiration rate of the leaf. The assessments took place on a sunny day between 10:30 and 11:30 AM, coinciding with the period when the light intensity reached saturation levels for photosynthesis^29^.

### Antioxidants

The evaluation of superoxide dismutase (SOD) activity involved investigating the suppression of nitro blue tetrazolium (NBT) reduction in the presence of riboflavin. An enzyme extract, NBT, riboflavin, and phosphate buffer constituted the reaction mixture. Subsequently, the change in absorbance at 560 nm was monitored after illuminating the mixture^30^. Peroxidase (POD) activity assessment relied on observing the oxidation of a suitable substrate such as guaiacol or o-dianisidine. The quantification was based on the increase in absorbance at a specific wavelength, indicating substrate oxidation^31^. Catalase (CAT) activity determination involved observing the enzyme's decomposition of hydrogen peroxide (H_2_O_2_). The reduction in absorbance at 240 nm due to H_2_O_2_ breakdown was noted^32^. Ascorbate peroxidase (APX) activity evaluation entailed monitoring the oxidation of ascorbate in the presence of H_2_O_2_^33^. The decrease in absorbance at a specific wavelength over time served as an indicator of APX activity. To quantify the content of malondialdehyde (MDA) thiobarbituric acid (TBA) method was used^34^.

### Electrolyte leakage

Fresh leaf pieces weighing approximately one gram were taken having a uniform size which was achieved by using a steel cylinder with a 1 cm diameter. We then supplemented each test tube containing the leaf fragments with 20 ml of deionized water. After that, we incubated the test tubes at 25 °C for 24 h to allow the electrolytes from the leaf tissues to diffuse into the water. Once the incubation period was over, we measured the electrical conductivity (EC1) of the water solution using a calibrated EC meter. Following this, we heated the test tubes in a water bath at 120 °C for 20 min to measure the second electrical conductivity (EC2)^35^.

$$\mathrm{Electrolyte \,Leakage }\left(\mathrm{\%}\right)=\left(\frac{{\text{EC}}1}{{\text{EC}}2}\right)\times 100$$

### N, P, and K shoot and root

A modified version of the micro-Kjeldahl technique as published by^36^ was used to estimate the nitrogen concentration. A flame photometer was used to test the potassium concentration. Furthermore, using a spectrophotometer and the yellow color technique^37^, the phosphorus concentration was measured at 420 nm.

### Statistical analysis

The significance of treatment combinations was determined using a two-way analysis of variance (ANOVA). Pairwise comparisons of treatments were conducted using the Tukey test, with a significance threshold set at p ≤ 0.05. Using OriginPro software from^38^, hierarchical cluster plots, cluster plot convex hull, and pearson correlation analysis were carried out to visualize data patterns.

### Study protocol must comply with relevant institutional, national, and international guidelines and legislation

Our experiment follows the with relevant institutional, national, and international guidelines and legislation.

## Results

**Figure 1:**
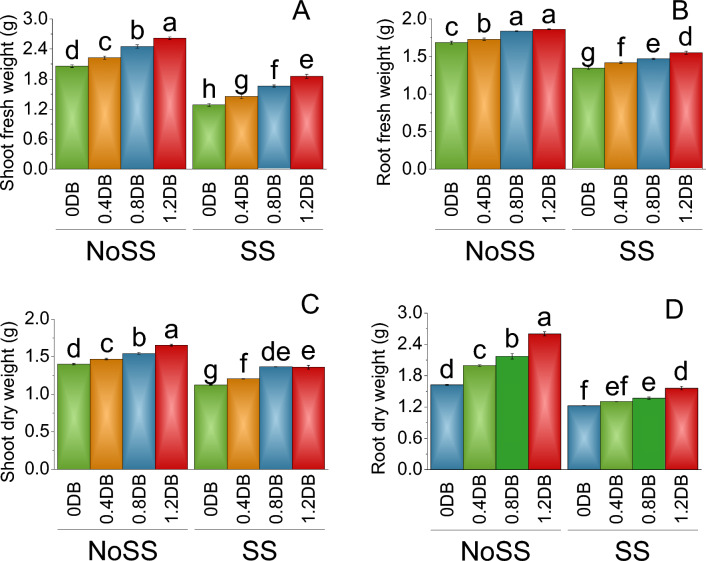
Impact of different levels of deashed biochar on shoot fresh weight (**A**), root fresh weight (**B**), shoot dry weight (**C**) and root dry weight (**D**) of fenugreek cultivated under no salinity stress (No SS) and salinity stress (SS). Bars are means of four replicates ± SE compared using Fisher’s LSD (p ≤ 0.05).

Treatment 0.4% deashed biochar showed a 2.23 g increase in the shoot fresh weight, representing a percentage increase of 7.53% over no salt stress. Further, the shoot fresh weight increased to 2.45 g in the 0.8% deashed biochar treatment, indicating a percentage increase of 16.02% under no salt stress. The largest increase in shoot fresh weight was observed in the 1.2% deashed biochar treatment, with a value of 2.62 g and a percentage increase of 21.32% under no salt stress. In salinity stress (SS), the shoot fresh weight was generally lower compared to the no salt stress. In 0% deashed biochar, the shoot fresh weight was 1.29 g. The 0.4% deashed biochar treatment resulted in a shoot fresh weight of 1.46 g, indicating a percentage increase of 11.68% related to the control group (SS). The largest increase was observed in the 1.2% deashed biochar, where the shoot fresh weight reached 1.86 g, showing a percentage increase of 30.82% over the control salt stress (Fig. 1A).

In the case of no SS plants, the root fresh weight increased from 1.69 g in the 0% deashed biochar to 1.73 g in the 0.4% deashed biochar, representing a percentage increase of 2.60% over the control. Further, the root fresh weight increased to 1.84 g in the 0.8% deashed biochar, indicating a percentage increase of 8.30% related to no salt stress. The largest increase in root fresh weight was observed in the 1.2% deashed biochar, with a value of 1.86 g and a percentage increase of 9.53% in no salt stress. In the control group (0% deashed biochar) of SS, the root fresh weight was 1.35 g. At 0.4% deashed biochar, root fresh weight was 1.42 g, indicating a percentage increase of 4.94% related to the control group. Similarly, the root fresh weight increased to 1.47 g in the 0.8% deashed biochar, representing a percentage increase of 8.33% than 0% deashed biochar in SS. The largest percentage increase was observed in the 1.2% deashed biochar, where the root fresh weight reached 1.55 g, showing a percentage increase of 13.06% under salt stress (Fig. 1B).

The root fresh weight increased to 1.47 g in the 0.4% deashed biochar, showing an increment of 4.59% over no SS. Furthermore, the shoot dry weight further increased to 1.55 g in the 0.8% deashed biochar treatment, signifying a percentage rise of 9.22% when related to the control group (NoSS). The most substantial enhancement in shoot dry weight was observed in the 1.2% deashed biochar treatment, reaching a value of 1.66 g, representing a percentage augmentation of 15.26% over the control group (NoSS). In the control group (0% deashed biochar ) in SS, 1.13 g of the shoot dry weight was recorded. The application of the 0.4% deashed biochar treatment resulted in a shoot dry weight of 1.21 g, denoting a percentage increase of 6.83% related to the SS (Fig. 1C).

Under no SS, the control group had a root dry weight of 1.71 g. When treated with 0.4% deashed biochar, the root dry weight increased to 2.00 g, indicating a 14.41% increase in root dry weight under no salt stress. Similarly, the 0.8% deashed biochar  resulted in a root dry weight of 2.17 g, representing an increase of 21.40% over no SS. The highest increase in root dry weight was observed with the 1.2% deashed biochar, reaching 2.60 g which showed a 34.39% increase over no SS. The application of 0.4% deashed biochar led to a significant increase in root dry weight to 1.21 g, showing a 14.23% increase over SS (Fig. 1D).

**Figure 2:**
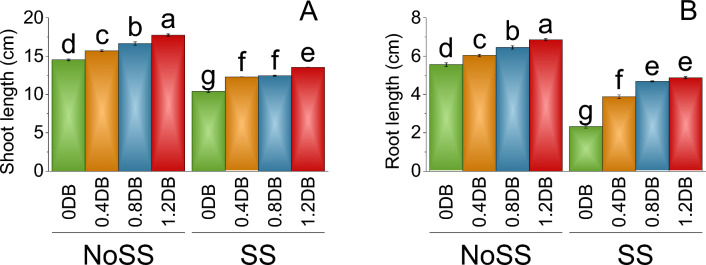
Impact of different levels of deashed biochar on shoot length (**A**) and root length (**B**) of fenugreek cultivated under no salinity stress (No SS) and salinity stress (SS). Bars are means of four replicates ± SE compared using Fisher’s LSD (p ≤ 0.05).

In the no SS (0% deashed biochar), the shoot length was 14.51 cm. However, at 0.4% deashed biochar, the shoot length increased to 15.70 cm, indicating a percentage increase of 7.58% compared to no salinity tress at 0% deashed biochar. Similarly, the shoot length for the 0.8% deashed biochar was 16.64 cm, showing a percentage increase of 12.79% than no SS. The 1.2% deashed biochar treatment resulted in a shoot length of 17.73 cm, representing an 18.15% increase compared to no SS 0% deashed biochar. In the SS treatment group, the shoot length without deashed biochar was 10.42 cm. When 0.4% deashed biochar was added, the shoot length increased to 12.29 cm, indicating a percentage increase of 15.23% compared to SS 0% deashed biochar (Fig. 2A).

Significant variation in the root length was observed at different treatments, in the absence of salinity stress (NoSS) and with no deashed biochar, the root length was 5.56 cm. When 0.4% deashed biochar was added under no SS, the root length increased to 7.19% over the control (0% deashed biochar). Further addition of 0.8% deashed biochar, showed a 13.84% increase in the root length at 6.45 cm and 6.86 cm at 1.2% deashed biochar, which represents an 18.96% increase in the root length over the control NoSS (0% deashed biochar). In the presence of SS and without deashed biochar, the root length was 2.32 cm. However, when 0.4% deashed biochar treatment was added to the SS, the root length significantly increased to 3.88 cm which shows a 40.24% increase over the control 0% deashed biochar under SS (Fig. 2B).

**Figure 3:**
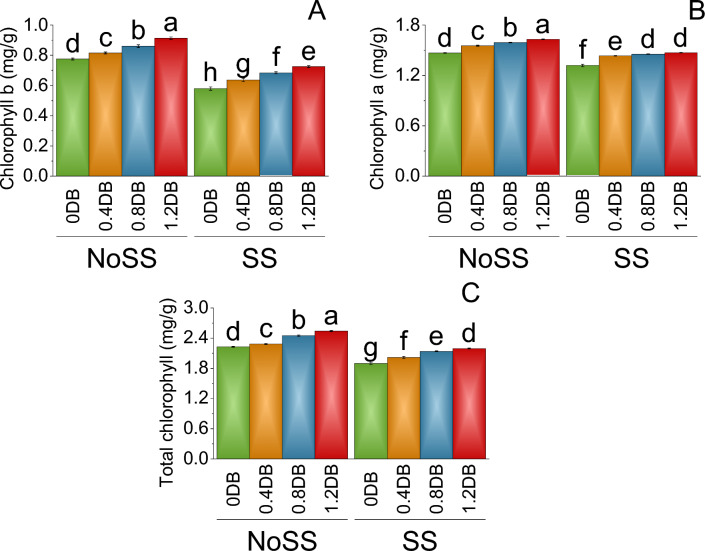
Impact of different levels of deashed biochar on chlorophyll *a* (**A**), chlorophyll *b* (**B**), and total chlorophyll (**C**) of fenugreek cultivated under no salinity stress (No SS) and salinity stress (SS). Bars are means of four replicates ± SE compared using Fisher’s LSD (p ≤ 0.05).

When there was no SS, a gradual increase in Chlorophyll *b* was observed with an increase in the applied dose of deashed biochar. The mean Chlorophyll *b* level at 0.4% deashed biochar under no SS was 0.82, representing a 4.91% increase compared to the baseline (0% deashed biochar). Similarly, at 0.8% deashed biochar there was a 9.88% increase, and at 1.2% deashed biochar, there was a significant 15.07% increase in Chlorophyll *b* levels over 0% deashed biocahar under no SS. On the other hand, in the control group 0% DB no salinity stress, chlorophyll *b* levels were 0.58. At 0.4% Deashed Biochar, there was a 9.02% increase in Chlorophyll *b* levels compared to 0% deashed biochar in SS (Fig. 3A).

In 0% deashed biochar under no salinity tress (NoSS), chlorophyll levels were 1.47 mg/g. The mean Chlorophyll *a* level at 0.4% deashed biochar (NoSS) was 1.55 mg/g, representing a 5.49% increase compared to the baseline 0% deashed biochar (NoSS). Similarly, at 0.8% deashed biochar treatment, there was a 7.70% increase, and at 1.2% deashed biochar, there was a significant 9.94% increase in Chlorophyll *b* levels over the control 0% deashed biochar under no SS. On the other hand, in the control group 0% deashed biochar under SS, chlorophyll levels were 1.32 mg/g. At 0.8% deashed biochar treatment, there was a 9.32% increase, and at 1.2% deashed biochar, there was a 10.34% increase in Chlorophyll *a* level then the control 0% deashed biochar under SS (Fig. 3B).

In no SS, the total chlorophyll content in the control group 0% deashed biochar was 2.23 mg/g. The 0.4% deashed biochar treatment showed a mean of 2.29 mg/g, resulting in a 2.47% increase in the total chlorophyll content over the control group 0% deashed biochar under no SS. Furthermore, the 0.8% deashed biochar treatment led to a mean total chlorophyll content of 2.45 mg/g, indicating a 9.07% increase compared to no SS control group 0% deashed biochar. The largest increase in the total chlorophyll content was observed in the 1.2% deashed biochar, with a mean of 2.54 mg/g, reflecting a 12.36% increase in total chlorophyll content to the control group 0% deashed biochar under no salinity. Under SS, compared to the control group (0% deashed biochar) with a mean of 1.90 mg/g, there was an increase in total chlorophyll levels due to the applied treatments. The main percentage increase was observed in the 1.2% deashed biochar treatment, with a mean of 2.20 mg/g, reflecting a 13.53% increase compared to the control group 0% deashed biochar under SS (Fig. 3C).

**Figure 4:**
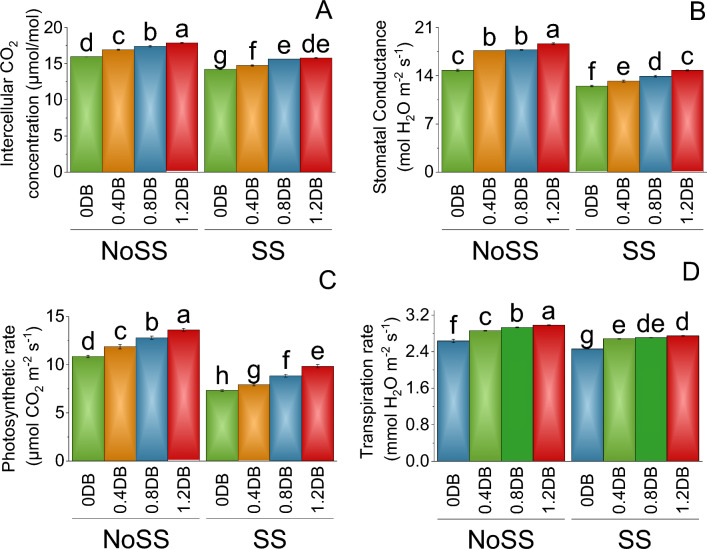
Impact of different levels of deashed biochar on intercellular CO_2_ concentration (**A**), stomatal conductance (**B**), photosynthetic rate (**C**) and transpiration rate (**D**) of fenugreek cultivated under no salinity stress (No SS) and salinity stress (SS). Bars are means of four replicates ± SE compared using Fisher’s LSD (p ≤ 0.05).

In no SS treatment, the mean stomatal CO_2_ concentration at 0% deashed biochar was 15.97. When a 0.4% deashed biochar was introduced, the stomatal CO_2_ concentration increased to 16.93, representing a 5.70% increase compared to the no SS. Further increases were observed at 0.8% deashed biochar treatment, resulting in a mean stomatal CO_2_ concentration of 17.40, indicating an 8.26% increase over the no SS. However, the 1.2% deashed biochar led to a mean stomatal CO_2_ concentration of 17.88, reflecting a significant 10.72% increase compared to the 0.8% deashed biochar under no SS. Under SS, the mean stomatal CO_2_ concentration at 0% deashed biochar was lower at 14.22. Conversely, the 0.4% deashed biochar treatment showed an increase in stomatal CO_2_ concentration to 14.75, indicating a 3.56% increase from the SS. The 0.8% deashed biochar further elevated the stomatal CO_2_ concentration to 15.64, representing a 9.06% increase compared to the SS (Fig. 4A).

In the no SS, stomatal conductance at 0% deashed biochar was 15.80. When the deashed biochar was introduced at the percentage of 0.4% deashed biochar, the stomatal conductance increased to 16.52, representing a 4.37% increase compared to the previous treatment of 0% deashed biochar. Further increasing the deashed biochar to 0.8% deashed biochar resulted in stomatal conductance of 17.52, indicating a 9.79% increase from the control 0% deashed biochar under SS. The highest deashed biochar treatment of 1.2% deashed biochar led to a mean stomatal conductance of 18.66, reflecting a significant 15.34% increase compared to the control group 0% deashed biochar (NoSS). Under the SS, treatment, the mean stomatal conductance at 0% deashed biochar was lower at 12.50. However, the introduction of a 0.4% deashed biochar treatment resulted in an increase in stomatal conductance to 13.21, indicating a 5.41% increase from the control group 0% deashed biochar under SS. The 0.8% Deashed Biochar treatment further elevated the stomatal conductance to 13.92, representing a 10.24% increase compared to the control 0% deashed biochar under SS (Fig. 4B).

In the absence of SS, the photosynthetic rate was higher. In the control group (0% deashed biochar), the photosynthetic rate was 10.86. The 0.4% deashed biochar showed a photosynthetic rate of 11.87, resulting in a percentage increase of 8.53% over the control group 0% deashed biochar under no SS. Furthermore, the 0.8% deashed biochar led to a photosynthetic rate of 12.81, indicating a 15.24% increase compared to the control group 0% deashed biochar under no SS. The higher percentage increase was observed in the 1.2% deashed biochar, photosynthetic rate was 13.61, reflecting a 20.26% increase from the control group 0% deashed biochar under no SS. Under salinity stress, compared to the control group (0% deashed biochar) with a photosynthetic rate of 7.33, there was an increase in the photosynthetic rate due to the applied treatments. The 0.4% deashed biochar treatment resulted in an improved photosynthetic rate of 7.93, representing a percentage increase of 7.48% over the control group 0% deashed biochar under SS (Fig. 4C).

In the absence of SS, the transpiration rate was higher. In the control group (0% deashed biochar), the transpiration rate was 2.64. The 0.4% deashed biochar treatment showed a transpiration rate of 2.86, resulting in a percentage increase of 7.79% over the no SS, 0% deashed biochar. Furthermore, the 0.8% deashed biochar treatment led to a transpiration rate of 2.93, indicating a 10.07% increase compared to the control group 0% deashed biochar under no SS. Under SS, compared to the control group (0% deashed biochar) with a transpiration rate of 2.46, there was an increase in the transpiration rate due to the applied treatments. The 0.8% deashed biochar led to a transpiration rate of 2.71, indicating a 9.14% increase over the control 0% deashed biochar under SS. The largest percentage increase was observed in the 1.2% deashed biochar, with a transpiration rate of 2.75, reflecting a 10.46% increase compared to the SS under 0% deashed biochar (Fig. 4D).

**Figure 5:**
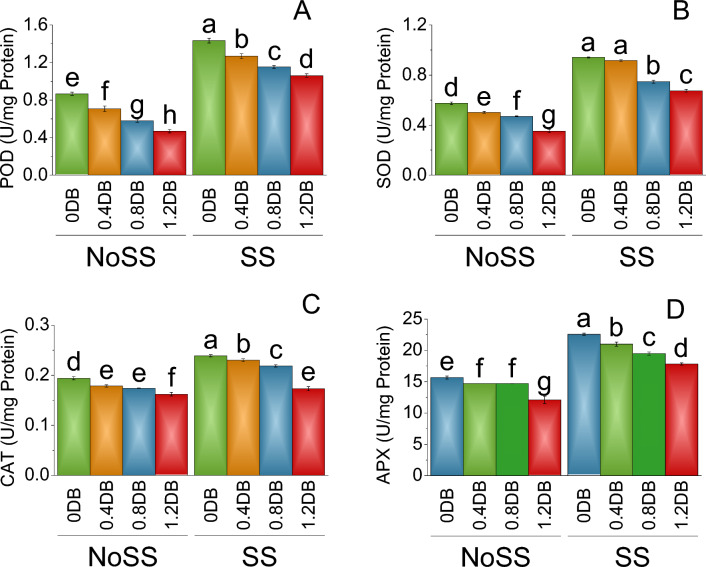
Impact of different levels of deashed biochar (DB) on POD (**A**), SOD (**B**), CAT (**C**) and APX (**D**) of fenugreek cultivated under no salinity stress (No SS) and salinity stress (SS). Bars are means of four replicates ± SE compared using Fisher’s LSD (p ≤ 0.05).

The significant effect of the treatments was also observed in peroxidase (POD) activity. In the no SS, the mean POD activity at 0% deashed biochar was 0.87 U/mg protein. When 0.4% Deashed Biochar treatment was introduced to the plant, the POD activity decreased to 0.71, representing an 18.44% decrease compared to the salinty stress. The 1.2% deashed biochar treatment, led to a POD activity of 0.47, reflecting a substantial 46.11% decrease compared to no salintiy stress. Under SS treatment, POD activity at 0% deashed biochar was higher at 1.43. However, the 0.4% deashed biochar treatment resulted in a decrease in POD activity to 1.27, indicating an 11.52% decrease from the SS. The 0.8% deashed biohcar treatment further lowered the POD activity to 1.15, representing a 19.55% decrease compared to the 0.4%% deashed biochar treatment and SS under 0% Deashed Biochar (Fig. 5A).

The significant impact of the treatments on superoxide dismutase (SOD) activity was also assessed. In no SS, SOD activity at 0% deashed biochar was recorded as 0.58. Upon the introduction of the 0.4% deashed boochar, the SOD activity slightly decreased to 0.50, exhibiting a 12.61% reduction compared to the no SS. Subsequently, the application of the 0.8% deashed biochar treatment resulted iin SOD activity of 0.47, signifying an 18.26% decrease compared to the no SS. The 1.2% deashed biochar treatment yielded SOD activity of 0.35, showed a significant 38.70% decrease relative to both the 0.8% deashed biochar treatment and no salinity stress. Under salinity stress, SOD activity at 0% deashed biochar was relatively higher at 0.94. However, the implementation of the 0.4% deashed biochar treatment caused a slight decline in SOD activity to 0.92, indicating a 2.66% decrease compared to the 0% deashed biochar control (SS). Furthermore, the 0.8% deashed biochar treatment led to a reduction in SOD activity to 0.75, which corresponded to a 20.48% decrease relative to both the 0.4% deashed biochar treatment under SS (Fig. 5B).

Catalase (CAT) activity at 0% deashed biochar was 0.19 under no SS. When 0.4% deashed biochar treatment was given to the plant, the CAT activity decreased slightly to 0.18, representing an 8.07% decrease compared to no SS. A further decrease in CAT activity of 0.17 was observed at 0.8% deashed biochar, indicating a 10.27% decrease over no SS. The 1.2% deashed biochar treatment led to CAT activity of 0.16, reflecting a significant 16.56% decrease compared to the both 0.8% deashed biochar under no SS. Under SS, CAT activity at 0% deashed biochar was higher at 0.24. However, at 0.4% deashed biochar resulted in a decrease in CAT activity to 0.23, indicating a 3.68% decrease from the control group (SS). The 0.8% deashed biochar further lowered the CAT activity to 0.22, representing an 8.45% decrease compared to the 0.4% deashed biochar treatment and control (SS) (Fig. 5C).

In SS treatment, the mean activity of ascorbate peroxidase (APX) at 0% deashed biochar was determined to be 15.67. The 0.4% deashed biochar treatment led to a slight reduction in APX activity, resulting in a mean value of 14.72, which represented a 6.05% decrease compared to no SS. Further analysis revealed that the application of the 0.8% deashed biochar treatment caused a subsequent decrease in APX activity to 14.69, signifying a 6.21% decrease from the previous treatment. The 1.2% deashed biochar treatment led to a significant decrease in APX activity to a mean value of 12.10, which reflected a substantial 22.79% decrease compared to both the 0.8% deashed biochar treatment and the control treatment at 0% deashed biochar under no SS. Under salinity stress treatment, APX activity at 0% deashed biochar was found to be higher, with a value of 22.58. However, the implementation of the 0.4% deashed biochar treatment caused a decline in APX activity to 20.99, indicating a 7.03% decrease compared to the no SS. The application of 0.8% deashed biochar further reduced APX activity to a mean value of 19.49, representing a 13.65% decrease relative to the 0.4% deashed biochar in SS (Fig. 5D).

**Figure 6:**
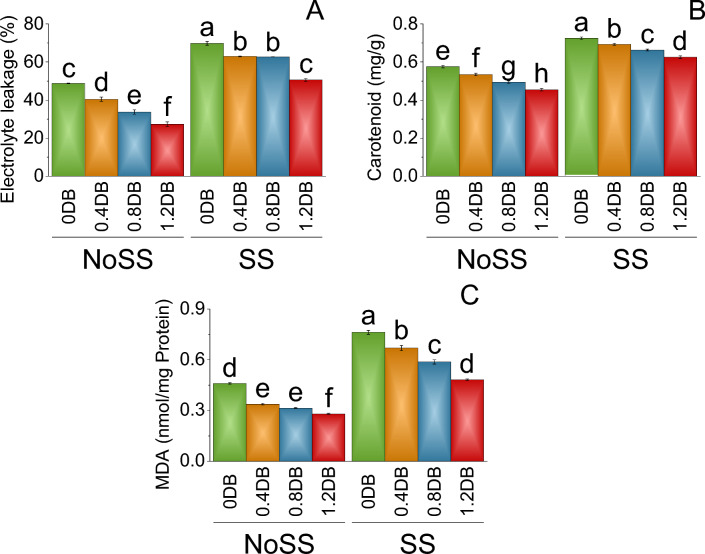
Impact of different levels of deashed biochar (% Deashed Biochar) on electrolyte leakage (**A**), carotenoid (**B**) and MDA (**C**) of fenugreek cultivated under no salinity stress (No SS) and salinity stress (SS). Bars are means of four replicates ± SE compared using Fisher’s LSD (p ≤ 0.05).

In no SS, electrolyte leakage at 0% deashed biochar was determined to be 48.76. The 0.4% deashed biochar led to the electrolyte leakage decreasing to 40.45, representing a 17.05% decrease compared to no SS. The 0.8% deashed biochar resulted in a mean electrolyte leakage of 33.74, indicating a substantial 30.81% decrease over the control (NoSS). The 1.2% deashed biochar led to a mean electrolyte leakage of 27.34, reflecting a significant 43.93% decrease compared to no SS. Under SS, electrolyte leakage at 0% deashed biochar was higher at 69.74. However, 0.4% of deashed biochar showed a decrease in electrolyte leakage to 62.92, indicating a 9.78% decrease related to SS. The 0.8% deashed biochar treatment further lowered the electrolyte leakage to 62.69, representing a 10.12% decrease compared to SS. Treatment 1.2% deashed biochar led to a mean electrolyte leakage of 50.64, showing a significant 27.38% decrease from the 0.8% deashed biochar treatment and over SS (Fig. 6A).

In no SS, the mean carotenoid content at 0% deashed biochar was quantified as 0.58. Introducing the 0.4% deashed biochar led to a slight reduction in carotenoid content, resulting in a value of 0.54, which represented a 6.96% decrease compared to the no salinity stres. Under SS, carotenoid content at 0% deashed biochar was comparatively higher at 0.73. However, in 0.4% deashed biochar, the carotenoid content decreased to 0.69, indicating a 4.48% decrease from the control (SS). Furthermore, the 0.8% deashed biochar treatment led to a reduced carotenoid content of 0.66, representing an 8.62% decrease compared to both the 0.4% deashed biochar treatment under SS (Fig. 6B).

In no SS, malondialdehyde (MDA) level at 0% deashed biochar was found as 0.46. The 0.8% deashed biochar showed in MDA level of 0.32, representing a 31.52% decrease under no SS. The 1.2% deashed biochar treatment led to a mean MDA level of 0.28, reflecting a significant 39.13% decrease compared to the control 0% deashed biochar under no SS. Under salinity stress, MDA level at 0% deashed biochar was higher at 0.76. However, the introduction of a 0.4% deashed biochar resulted in a decrease in MDA levels to 0.67, indicating a 12.13% decrease from the salinity stress. The 0.8% deashed biochar further reduced the MDA level to 0.59, representing a 22.95% decrease compared to the 0.4% deashed biochar treatment under SS. The 1.2% deashed biochar led to MDA level of 0.48, showing a 36.72% decrease from the control (SS) (Fig. 6C).

**Figure 7:**
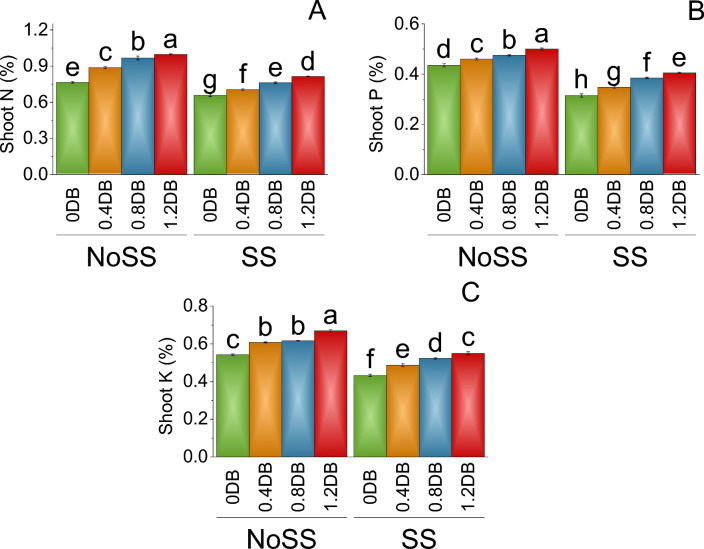
Impact of different levels of deashed biochar on shoot N (**A**), shoot P (**B**) and shoot K (**C**) of fenugreek cultivated under no salinity stress (No SS) and salinity stress (SS). Bars are means of four replicates ± SE compared using Fisher’s LSD (p ≤ 0.05).

In the no SS, at 0% deashed biochar, shoot N content was determined to be 0.77%. When the 0.4% deashed biochar treatment was introduced, the shoot N content increased to 0.89%, representing a 13.80% rise compared to no SS. Furthermore, the application of the 0.8% deashed biochar resulted in a mean shoot N content of 0.97%, indicating a substantial 20.93% increase from the no SS. Treatment with 1.2% deashed biochar led to a mean shoot N content of 1.00%, reflecting a significant 23.31% increase compared to both the 0.8% deashed biochar treatment and the control at 0% deashed biochar (NoSS). Under SS, the mean shoot N content at 0% deashed biochar was slightly lower at 0.66%. However, when the 0.4% deashed biochar was applied, the shoot N content increased to 0.71%, signifying a 6.74% increase compared to the control treatment (SS). The 0.8% deashed biochar treatment further elevated the shoot N content to 0.76%, representing a 13.77% increase relative to the 0.4% deashed biochar and the control (SS). Treatment with 1.2% deashed biochar resulted in a mean shoot N content of 0.82%, indicating a 19.33% increase from both the 0.8% deashed biochar and the SS (Fig. 7A).

In the no SS at 0% deashed biochar, the mean shoot P content was determined to be 0.44%. When the 0.4% deashed biochar treatment was applied, the shoot P content increased to 0.46%, representing a 5.43% increase compared to the control treatment (NoSS). Furthermore, the application of the 0.8% deashed biochar treatment resulted in a mean shoot P content of 0.48%, indicating an 8.42% increase from the control (NoSS). Under SS, the mean shoot P content at 0% deashed biochar was lower at 0.32%. However, when the 0.4% deashed biochar treatment was applied, the shoot P content increased to 0.35%, signifying a 9.35% increase from the SS. The 0.8% deashed biochar treatment further elevated the shoot P content to 0.39%, representing an 18.18% increase compared to the 0.4% deashed biochar treatment under SS (Fig. 7B).

In no SS at 0% deashed biochar, the mean shoot K content was determined to be 0.54%. In 0.4% deashed biochar, the shoot K content increased to 0.61%, representing a 10.77% increase compared to no SS. Furthermore, the application of the 0.8% deashed biochar treatment resulted in a mean shoot K content of 0.62%, indicating a 12.04% increase from no SS. The 1.2% deashed biochar led to shoot K content of 0.67%, reflecting a significant 19.00% increase compared to the 0.8% deashed biochar and the control at 0% deashed biochar (NoSS). Under SS conditions, the mean shoot K content at 0% deashed biochar was lower at 0.43%. However, when the 0.4% deashed biochar was applied, the shoot K content increased to 0.49%, signifying an 11.13% increase from SS. The 0.8% deashed biochar treatment further elevated the shoot K content to 0.52%, representing a 17.27% increase compared to the SS (Fig. 7C).

**Figure 8:**
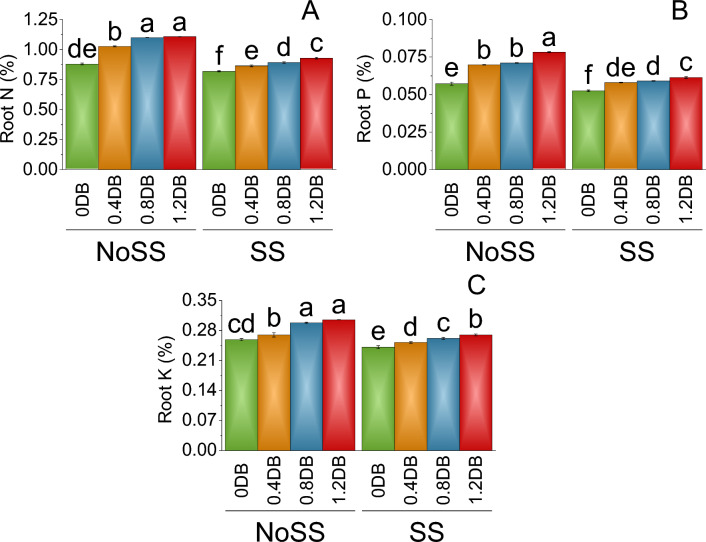
Impact of different levels of deashed biochar on root N (**A**), root P (**B**) and root K (**C**) of fenugreek cultivated under no salinity stress (No SS) and salinity stress (SS). Bars are means of four replicates ± SE compared using Fisher’s LSD (p ≤ 0.05).

The nitrogen (N) content in the roots (%) was determined to be 0.88% in no SS at 0% deashed biochar. Introducing the 0.4% deashed biochar treatment led to an increase in root N content to 1.03%, corresponding to a 14.28% rise compared to no SS. Subsequently, applying the 0.8% deashed biochar treatment resulted in a mean root N content of 1.10%, representing a 20.01% increase from no SS. The highest root N content of 1.11% was observed in the 1.2% deashed biochar treatment, exhibiting a significant 20.60% increase compared to the control at 0% deashed biochar (NoSS). In SS, root N content at 0% deashed biochar was relatively lower at 0.82%. Further application of the 0.8% deashed biochar treatment led to a mean root N content of 0.89%, demonstrating an 8.05% increase compared to SS. The highest root N content of 0.93% was observed in the 1.2% deashed biochar treatment, showing an 11.54% increase from both the 0.8% deashed biochar treatment and the control (SS) (Fig. 8A).

In no SS, at 0% deashed biochar, the mean root P content was found to be 0.06%. Upon the introduction of the 0.4% deashed biochar, the root P content increased to 0.07%, representing a 17.91% increase under no SS. Similarly, the application of the 0.8% deashed biochar treatment resulted in a mean root P content of 0.07%, indicating a 19.33% increase from no SS. The highest root P content of 0.08% was observed in the 1.2% deashed biochar, reflecting a significant 26.75% increase compared to the control at 0% deashed biochar (NoSS). Under salinity, the mean root P content at 0% deashed biochar was lower, measuring 0.05%. However, with the application of the 0.4% deashed biochar treatment, the root P content increased to 0.06%, signifying a 9.55% increase under SS. The 1.2% deashed biochar resulted in a similar mean root P content of 0.06%, indicating a 14.41% increase from the control treatment in salinity-stressed conditions (Fig. 8B).

In no SS at 0% deashed biochar, the mean root N content was found to be 0.26%. When the 0.4% deashed biochar treatment was applied, there was a slight increase in the root N content to 0.27%, representing a 4.09% increase under no SS. Furthermore, the application of the 0.8% deashed biochar resulted in a mean root N content of 0.30%, indicating a 13.11% increase under no SS. The 1.2% deashed biochar further elevated the root N content to 0.31%, reflecting a significant 15.19% increase related to the control at 0% Deashed Biochar under no SS. Under SS conditions, root N content at 0% deashed biochar was lower, measuring 0.24%. The 0.8% deashed biochar treatment further raised the root N content to 0.26%, representing a 7.81% increase over the control (SS). Treatment with 1.2% deashed biochar resulted in a mean root N content of 0.27%, indicating a 10.57% increase from the control treatment in salinity-stressed conditions (Fig. 8C).

**Figure 9:**
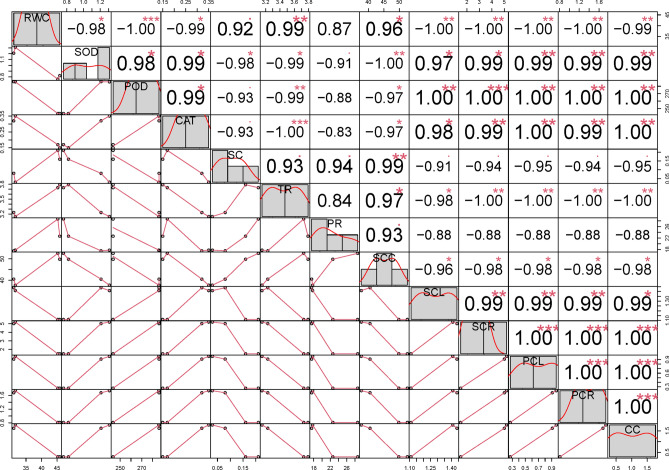
Pearson correlation chart of measured parameters.

The study found positive correlations between shoot fresh weight and parameters such as shoot dry weight, root fresh weight, chlorophyll content, total chlorophyll, and photosynthetic rate, while negative correlations were observed with electrolyte leakage and various enzymatic activities. Salinity stress showed a positive correlation with shoot nitrogen content but negative correlations with root and shoot fresh and dry weights, shoot length, chlorophyll content, and total chlorophyll. Additionally, SS correlated positively with electrolyte leakage and peroxidase activity (Fig. 9).

**Figure 10:**
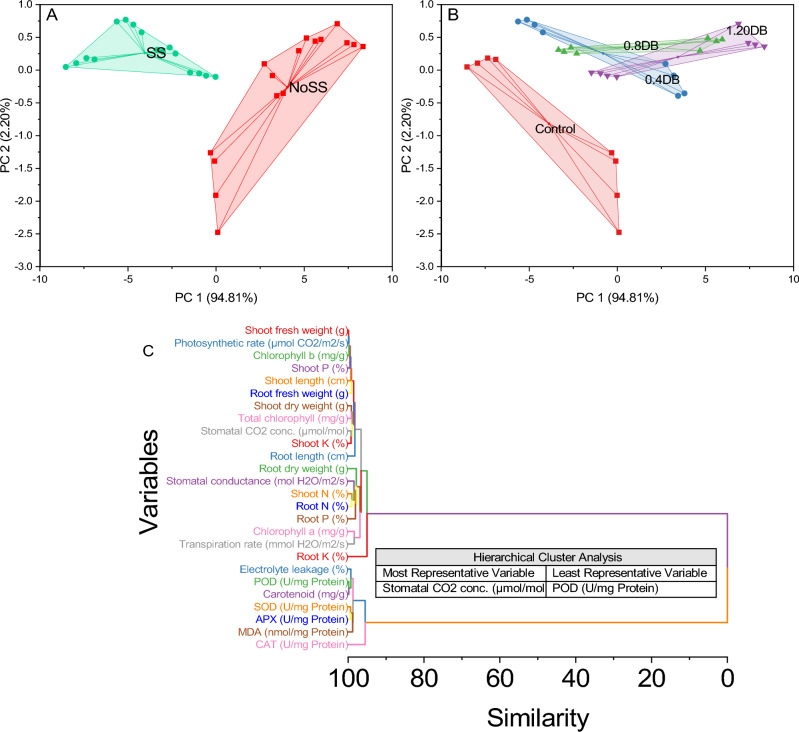
Cluster plot with convex hull for salinity (**A**), deashed biochar (**B**) and hierarchical cluster plot for studied attributes (**C**).

A cluster plot with convex hull was employed to evaluate the impact of treatments and salinity stress on various attributes, focusing on principal components PC1 and PC2. PC1, explaining 94.81% of the variance, revealed distinct patterns among treatment groups, with the 0% deashed biochar group clustered in the lower region and 1.2% Deashed Biochar in the upper. PC2 scores further highlighted differences, with 0% deashed biochar exhibiting lower scores and 1.2% deashed biochar higher scores. The hierarchical cluster plot identified internal stomatal concentration as the most representative variable, while peroxidase (POD) was the least representative. This analysis provides insights into the complex relationships between treatments, SS, and plant attributes (Fig. 10).

## Discussion

This study aimed to assess the impact of deashed biochar under both saline and non-saline conditions on the generation of free radicals in plants. In salinity stress, free radicals, highly reactive molecules, can inflict harm on cellular components such as proteins, DNA, and cells, thereby contributing to overall plant damage^39,40^. Antioxidants play a crucial role in mitigating this damage by neutralizing free radicals. They achieve this by donating electrons to stabilize and neutralize these reactive molecules^39^. Under saline stress conditions, there was increase in electrolyte leakage and a decrease in carotenoids compared to conditions without salinity stress (Fig. 6). These observations indicate that the plants experienced salinity stress. The high production of antioxidants, such as peroxidase, superoxidase, catalase, and ascorbic acid, in response to salinity stress (Fig. 5) underscores the generation of reactive oxygen species. However, application of deashed biochar resulted in a reduction in antioxidant production. This decline was more pronounced with higher deashed biochar application rates (Fig. 5). The correlation between reduced antioxidant production and decreased electrolyte leakage with deashed biochar application (Fig. 6) suggests a protective effect against salinity-induced stress.

The application of deashed biochar significantly alleviated salinity stress, leading to increased uptake of nitrogen, phosphorus, and potassium in both the shoot and root of plants, (Figs. 7 and 8). When deashed biochar is incorporated into the soil, it acts as a reservoir for these essential nutrients, preventing their leaching and making them available for plant uptake. This increased nutrient availability is particularly beneficial under salinity stress, where the uptake of N, P, and K may be hindered due to the adverse effects of high salt levels on root functioning^69^. This enhanced nutrient absorption subsequently promoted greater fresh and dry weights in both shoot and root, along with increased shoot and root lengths (Figs. 1 and 2). Farouk and Al-Huqail ^41^ found 42.49%, 899.06%, and 96.0% increase in plant height, shoot fresh weight, and shoot dry weight, respectively with application of biochar in saline condition (500 ppm NaCl). The heightened vegetative growth facilitated by the presence of deashed biochar also facilitated an increased uptake of water. Akhtar et al.^12^ found increased in the water use with application of biochar at all salinity levels of 0, 25 and 50 mM NaCl. Furthermore, the introduction of biochar has the potential to increase the soil's water-holding capacity, especially in situations with reduced irrigation, as found by Akhtar et al.^42^. This enhancement in water retention is expected to alleviate salt-induced osmotic stress and ion toxicity in plants, attributable to a dilution effect. The high uptake of water might be contributed to the synthesis of chlorophyll a, b, and total chlorophyll (Fig. 3). The elevated chlorophyll content further resulted in a higher photosynthetic rat (Fig. 4). Farouk and Al-Huqail^41^ found 0.89% increase in total chlorophyll with application of biochar under salinity stress. Moreover, the ample water availability in conjunction with the presence of biochar led to increased stomatal conductance and internal stomatal CO_2_ concentration (Fig. 4). Akhtar et al.^12^ observed elevated midday leaf water potential, increased shoot biomass, enhanced root length and volume, higher tuber yield, and a reduction in abscisic acid (ABA) concentration in both leaf and xylem sap when biochar was applied. These effects were noted across three different salinity levels (0, 25, and 50 mM NaCl solutions) compared to their corresponding non-biochar controls. These multifaceted effects of deashed biochar highlight its potential as a valuable soil amendment for improving nutrient uptake, chlorophyll content, and stress tolerance in plants grown under salinity stress conditions.

## Conclusion

The utilization of deashed biochar can alleviate the adverse impacts of salinity on fenugreek. It notably enhanced phosphorus and potassium concentrations in both root and shoot tissues, thereby promote fenugreek growth attributes. Furthermore, improvement in phosphorus and potassum concentrations by deashed biochar played a crucial role in regulation of antioxidants, that effectively mitigate the oxidative stress induced by salinity. The promising outcomes of this study suggest that strategically applying de-ashed biochar at a rate of 1.2% holds considerable potential for significantly improving fenugreek crop productivity in saline soils. Growers are suggested to incorporate de-ashed biochar at a rate of 1.2% to achieve better crop yields in salt-affected soils.

## Data Availability

All data generated or analysed during this study are included in this published article.

## References

[1] Ashraf F, Chen Y (2023). Synergistic effects of biochar and arbuscular mycorrhizal fungi on enhancing *Elymus elymoides* growth in saline coastal soil. Pak. J. Bot..

[2] Singh G (2009). Salinity-related desertification and management strategies: Indian experience. Land Degrad. Dev. [Internet].

[3] Shahid, S. A., Zaman, M. & Heng, L. *Soil Salinity: Historical Perspectives and a World Overview of the Problem. in Guideline for Salinity Assessment, Mitigation and Adaptation Using Nuclear and Related Techniques* (eds. Zaman, M., Shabbir, A. & Heng, S. L.) 43–53 (Springer International Publishing, 2018). 10.1007/978-3-319-96190-3_2.

[4] Childs SW, Hanks RJ (1975). Model of soil salinity effects on crop growth. Soil Sci. Soc. Am. J..

[5] Hailu, B. Impacts of Soil Salinity/Sodicity on Soil-Water Relations and Plant Growth in Dry Land Areas: A Review. in Limitations to Efficient Water Use in Crop Production (eds. Taylor, H. M., Jordan, W. R. & Sinclair, T. R.). 10.2134/1983.limitationstoefficien (2021). 10.7176/JNSR/12-3-01.

[6] Munns R, Gilliham M (2015). Salinity tolerance of crops—What is the cost?. New Phytol..

[7] Lacerda, C. F. et al. Soil salinization and maize and cowpea yield in the crop rotation system using saline waters. *Eng. Agrícola* **31**, 663–675 (2011).

[8] Guangming L, Xuechen Z, Xiuping W, Hongbo S, Jingsong Y, Xiangping W (2017). Soil enzymes as indicators of saline soil fertility under various soil amendments. Agric. Ecosyst. Environ..

[9] Das P, Nutan KK, Singla-Pareek SL, Pareek A (2015). Understanding salinity responses and adopting ‘omics-based’ approaches to generate salinity tolerant cultivars of rice. Front. Plant Sci..

[10] Lehmann, J. & Joseph, S. Biochar for Environmental Management. Taylor and Francis (Routledge, 2009). 10.4324/9781849770552.

[11] Jabborova, D., Ziyadullaeva, N., Enakiev, Y., Narimanov, A., Dave, A., Sulaymanov, K. *et al*. Growth of spinach as influenced by biochar and *Bacillus endophyticus* IGPEB 33 in drought condition. *Pak. J. Bot*. **55**, 53–59 (2023).

[12] Akhtar SS, Andersen MN, Liu F (2015). Biochar mitigates salinity stress in potato. J. Agron. Crop Sci..

[13] Kul R, Arjumend T, Ekinci M, Yildirim E, Turan M, Argin S (2021). Biochar as an organic soil conditioner for mitigating salinity stress in tomato. Soil Sci. Plant Nutr..

[14] Kanwal S, Ilyas N, Shabir S, Saeed M, Gul R, Zahoor M (2018). Application of biochar in mitigation of negative effects of salinity stress in wheat ( * Triticum *
* aestivum * L.). J. Plant Nutr. [Internet].

[15] Sun K, Kang M, Zhang Z, Jin J, Wang Z, Pan Z (2013). Impact of deashing treatment on biochar structural properties and potential sorption mechanisms of phenanthrene. Environ. Sci. Technol..

[16] Zhang P, Sun H, Ren C, Min L, Zhang H (2018). Sorption mechanisms of neonicotinoids on biochars and the impact of deashing treatments on biochar structure and neonicotinoids sorption. Environ. Pollut..

[17] Chen W, Wei R, Yang L, Yang Y, Li G, Ni J (2019). Characteristics of wood-derived biochars produced at different temperatures before and after deashing: Their different potential advantages in environmental applications. Sci. Total Environ..

[18] Ibrahim MM, Hu K, Tong C, Xing S, Zou S, Mao Y (2020). De-ashed biochar enhances nitrogen retention in manured soil and changes soil microbial dynamics. Geoderma.

[19] Abid M, Danish S, Zafar-ul-Hye M, Shaaban M, Iqbal MM, Rehim A (2017). Biochar increased photosynthetic and accessory pigments in tomato (*Solanum **lycopersicum* L.) plants by reducing cadmium concentration under various irrigation waters. Environ. Sci. Pollut. Res..

[20] Gee, G.W. & Bauder, J.W. *Particle-size analysis. In Methods of Soil Analysis. Part 1 Physical and Mineralogical Methods*. 2nd ed. 383–411 (1986).

[21] McLean EO, Page AL (1982). Soil pH and lime requirement. Methods of Soil Analysis: Part 2 Chemical and Microbiological Properties.

[22] Rhoades, J.D. *Salinity: Electrical Conductivity and Total Dissolved Solids. Methods of Soil Analysis, Part 3: Chemical Methods*.417–435 (2018).

[23] Walkley A (1935). An examination of methods for determining organic carbon and nitrogen in soils. J. Agric. Sci..

[24] Bremner JM, Mulvaney CS, Page AL, Miller RH, Keeney DR (1982). Nitrogen–total. Methods of Soil Analysis Part 2 Chemical and Microbiological Properties.

[25] Kuo, S. Phosphorus. In *Methods of Soil Analysis Part 3: Chemical Methods* (eds Sparks, D.L., Page, A.L., Helmke, P.A., Loeppert, R.H., Soltanpour, P.N., Tabatabai, M.A. *et al*.). 869–919 (SSSA/Wiley, 2018).

[26] Pratt, P.F. Potassium. In *Methods of Soil Analysis, Part 2: Chemical and Microbiological Properties* (ed. Norman, A.G.). 1022–1030 (Wiley, 2016).

[27] Boutraa T, Akhkha A, Al-Shoaibi AA, Alhejeli AM (2010). Effect of water stress on growth and water use efficiency (WUE) of some wheat cultivars (*Triticum durum*) grown in Saudi Arabia. J. Taibah Univ. Sci..

[28] Arnon DI (1949). Copper enzymes in isolated chloroplasts. Polyphenoloxidase in *Beta vulgaris*. Plant Physiol..

[29] Nazar R, Khan MIR, Iqbal N, Masood A, Khan NA (2014). Involvement of ethylene in reversal of salt-inhibited photosynthesis by sulfur in mustard. Physiol. Plant.

[30] Durak, I., Yurtarslanl, Z., Canbolat, O. & Akyol, Ö. A methodological approach to superoxide dismutase (SOD) activity assay based on inhibition of nitroblue tetrazolium (NBT) reduction. *Clin. Chim. Acta*. **214**, 103–104. https://linkinghub.elsevier.com/retrieve/pii/000989819390307P (1993). 10.1016/0009-8981(93)90307-p 8453769

[31] Cakmak I, Strbac D, Marschner H (1993). Activities of hydrogen peroxide-scavenging enzymes in germinating wheat seeds. J. Exp. Bot..

[32] Aebi, H. Catalase in vitro. In *Oxygen Radicals in Biological Systems: Methods in Enzymology *(ed Packer, L.). 121–126 (Elsevier BV, 1984). 10.1016/s0076-6879(84)05016-3 6727660

[33] Nakano Y, Asada K (1981). Hydrogen peroxide is scavenged by ascorbate-specific peroxidase in spinach chloroplasts. Plant Cell Physiol..

[34] Hernández JA, Almansa MS (2002). Short-term effects of salt stress on antioxidant systems and leaf water relations of pea leaves. Physiol. Plant [Internet].

[35] Lutts S, Kinet JM, Bouharmont J (1996). NaCl-induced senescence in leaves of rice (*Oryza sativa* L.) cultivars differing in salinity resistance. Ann. Bot..

[36] Steyermark AL, McGee BE (1961). Progress in elemental quantitative organic analysis: 1960. Microchem. J..

[37] Olsen, S.R., Sommers, L.E., Page, A.L. *et al*. *Methods of Soil Analysis. Part 2*. 403–30 (1982)

[38] OriginLab Corporation. *OriginPro*. (OriginLab, 2021).

[39] Apel K, Hirt H (2004). Reactive oxygen species: Metabolism, oxidative stress, and signal transduction. Annu. Rev. Plant.

[40] Miller G, Suzuki N, Ciftci-Yilmaz S, Mittler R (2010). Reactive oxygen species homeostasis and signalling during drought and salinity stresses. Plant Cell Environ..

[41] Farouk S, Al-Huqail AA (2022). Sustainable biochar and/or melatonin improve salinity tolerance in borage plants by modulating osmotic adjustment, antioxidants, and ion homeostasis. Plants..

[42] Akhtar SS, Li G, Andersen MN, Liu F (2014). Biochar enhances yield and quality of tomato under reduced irrigation. Agric. Water Manag..

